# Mining the deep Red-Sea brine pool microbial community for anticancer therapeutics

**DOI:** 10.1186/s12906-019-2554-0

**Published:** 2019-06-20

**Authors:** Luke Esau, Guishan Zhang, Sunil Sagar, Ulrich Stingl, Vladimir B. Bajic, Mandeep Kaur

**Affiliations:** 1 King Abdullah University of Science and Technology (KAUST), Computational Bioscience Research Center (CBRC), Thuwal, Jeddah, 23955-6900 Saudi Arabia; 2 Key Laboratory of Microbial Resources Collection and Preservation, Ministry of Agriculture, Institute of Agricultural Resources and Regional Planning, Chinese Academy of Agricultural Sciences, Beijing, 100081 People’s Republic of China; 3 King Abdullah University of Science and Technology, Red Sea Research Center, Thuwal, Jeddah, 23955-6900 Saudi Arabia; 4 Department of Microbiology and Cell Science, Institute of Food and Agricultural Sciences, University of Florida, UF/IFAS Fort Lauderdale Research and Education Center, Davie, FL 33314 USA; 5 School of Molecular and Cell Biology, University of the Witwatersrand, Johannesburg, 2050 South Africa

**Keywords:** Deep Red-Sea, Triple negative breast cancer, Brine pools, Anticancer

## Abstract

**Background:**

Microbial species in the brine pools of the Red Sea and the brine pool-seawater interfaces are exposed to high temperature, high salinity, low oxygen levels and high concentrations of heavy metals. As adaptations to these harsh conditions require a large suite of secondary metabolites, these microbes have a huge potential as a source of novel anticancer molecules.

**Methods:**

A total of 60 ethyl-acetate extracts of newly isolated strains from extreme environments of the Red-Sea were isolated and tested against several human cancer cell lines for potential cytotoxic and apoptotic activities.

**Results:**

Isolates from the Erba brine-pool accounted for 50% of active bacterial extracts capable of inducing 30% or greater inhibition of cell growth. Among the 60 extracts screened, seven showed selectivity towards triple negative BT20 cells compared to normal fibroblasts.

**Conclusion:**

In this study, we identified several extracts able to induce caspase-dependent apoptosis in various cancer cell lines. Further investigations and isolation of the active compounds of these Red Sea brine pool microbes may offer a chemotherapeutic potential for cancers with limited treatment options.

## Background

The hallmarks of cancer, which categorises the survival and proliferative mechanisms of cancer cells, was first described by Hanahan and Weinberg [1]. Although these aspects of cancer are well studied, and even with the advances in personalized medicine for patient tailored treatment, there still exists cancer forms for which limited treatment options are available. Breast cancer, the most prevalent cancer in women globally, has an estimated 1.67 million cases diagnosed in 2012 [2]. Women of Arab [3] and African descent [4] present breast cancer at an earlier age, have more aggressive tumour types with a high prevalence of triple negative breast cancer. Penta-negative tumours (i.e., negative for the estrogen and progesterone receptors, EGFR, HER2, and cytokeratin 5/6) have also been reported in Saudi women [5]. In our previous seminal investigations [6, 7], we reported the anticancer potential of extracts obtained from brine-pool microbes. Most of these extracts were active against estrogen receptor positive (ER+) breast cancer cells. Since the triple-negative type of breast cancer is a more aggressive form and as currently no therapies are available, we aimed to identify new sources of anticancer compounds that can pave the way to develop novel therapies for triple negative breast cancer.

Approximately 60 % of current anticancer therapeutics are derived from natural products, including, for example, marine-derived compounds such as cytarabine, trabectedin, eribulin, and dolastatins [8]. A review by Agrawal et al. [9] described nonribosomal peptides isolated from marine microbes having anticancer activity while in a research article Neelam et al. [10] discovered a marine halo-alkaliphilic bacteria species possessing anti breast cancer activity. This evidence supports the pursuit of mining marine environments for the discovery of new anticancer agents. Twenty-five (mostly) anaerobic deep-sea brine pools with extremely high salt concentrations have been reported in the Red Sea. Multi-extremophilic microbes that inhabit these environments are not only adapted to high salinity (4–26%), but also to elevated temperature, low oxygen concentrations, and high concentrations of heavy metals [11–14]. These extreme marine environmental conditions favour the production of secondary metabolites and thus potentially unique and potent natural compounds. Extremophilic marine bacterial species from these environments, therefore, present a unique opportunity for discovering novel anticancer compounds [6, 7] that address the ever-changing need for improved chemotherapeutic drugs.

This study reports on anticancer activity of extracts from bacterial isolates from different habitats around brine pools within the Red Sea. We screened a total of 60 extracts against seven cell lines representing colorectal carcinoma, fibrosarcoma, breast carcinoma, cervical carcinoma, neuroblastoma, and normal cell lines. After initial screening, active extracts (> 30% growth inhibition) were selected for testing in selected cell lines to investigate if apoptotic activities were moderated by caspases (executors of apoptotic cell death).

## Methods

### Field sampling

The inoculum for microbial isolations were collected during a cruise between Oct 16 and Nov 3 2011 as described in Sagar et al. [7].

### Source of bacterial isolates

Sixty bacterial strains were isolated from different habitats in or around the brine pools (Table 1). While the deep-sea brine pool habitats differ in their physicochemical characteristics [15], several studies showed that all of these environments harbour high microbial diversity and biomass.

**Table 1:** Identification of microbial stains. Taxonomic identification based on 16S rRNA gene analysis and the source of inoculum for 60 microbial strains

Name	Source	Salinity (w/v)	Closest relative	Similarity (16S rRNA genes)	Accession no. of the strains	Accessionno. of the closest relatives
SB9	Discovery interface	25%	*Haloprofundus marisrubri*	100%	KJ999759	FN594944
SB3	Discovery interface	25%	*Haloferax prahovense*	97%	KJ999758	NR_028165
SB29	Discovery interface	25%	*Haloferax larsenii*	98%	KJ999757	NR_028209
SA10	Kebrit brine	26%	*Haloferax prahovense*	99%	MG563761	NR_028165
ZGT108	Erba interface	10%	*Ruegeria profundi*	100%	KP726355	NR_029197
ZGT114	Erba interface	10%	*Microbulbifer salipaludis*	98%	KP726357	NR_025232
ZGT118	Erba interface	10%	*Ruegeria marisrubri*	100%	KP726356	NR_029197
SJ5A-1	Erba interface	10%	*Ponticoccus marisrubri*	97%	KP726358	NR_044174
SJ5B	Erba interface	10%	*Ponticoccus litoralis*	99%	MG764545	NR_044174
XI10	Erba interface	10%	*Pseudoalteromonas mariniglutinosa*	99%	MG768922	NR_028992
H106	Erba interface	10%	*Idiomarina zobellii*	99%	MG768917	NR_024892
1	Kebrit brine	26%	*Halomonas axialensis*	99%	MG768918	NR_027219
2	Kebrit interface	20%	*Halomonas salina*	99%	MG768925	NR_042050
3	Kebrit interface	20%	*Marinimicrobium haloxylanilyticum*	99%	MG768919	GQ920839
4	Kebrit interface	20%	*Halobacillus kuroshimensis*	99%	MG768920	NR_041262
5	Erba interface	10%	*Chromohalobacter israelensis*	99%	MG768921	NR_025431
6	Erba sediment	18%	*Alteromonas halophila*	99%	MG768928	EU583725
7	Erba sediment	18%	*Halomonas taeanensis*	98%	MG768923	NR_043087
8	Erba sediment	18%	*Halobacillus locisalis*	99%	MG768924	NR_025715
9	Erba interface	10%	*Alteromonas macleodii*	99%	MG768926	Y18228
10	Erba interface	10%	*Salinivibrio costicola.*	99%	MG768927	NR_028703
11	Erba interface	10%	*Halomonas denitrificans*	99%	MG768930	NR_042491
12	Erba interface	10%	*Pontibacillus marinus*	99%	MG768932	NR_043011
13	Nereus interface	10%	*Pseudoalteromonas mariniglutinosa*	98%	MG768936	NR_028992
14	Nereus interface	10%	*Pseudoalteromonas flavipulchra*	99%	MG768933	NR_025126
15	Nereus interface	10%	*Salinivibrio sharmensis*	99%	MG768935	AM279734
16	Nereus interface	10%	*Halomonas hamiltonii*	100%	MG768937	AM941396
17	Nereus interface	10%	*Salinicola salarius*	99%	MG768934	NR_042490
18	Discovery interface	15%	*Alteromonas macleodii*	98%	MG768957	Y18228
19	Discovery interface	15%	*Halomonas halophila*	99%	MG768941	NR_042697
20	Discovery interface	15%	*Alteromonas macleodii*	97%	MG768929	AM885870
21	Discovery interface	15%	*Pontibacillus chungwhensis*	98%	MG768940	NR_025812
22	Nereus interface	10%	*Salinicola salarius*	98%	MG768944	NR_042490
23	Nereus interface	10%	*Zunongwangia profunda*	99%	MG768951	NR_043986
24	Nereus interface	10%	*Marinobacter flavimaris*	99%	MG768954	NR_025799
25	Nereus interface	10%	*Chromohalobacter marismortui*	99%	MG768956	X87222
26	Kebrit interface	20%	*Salinivibrio proteolyticus*	99%	MG768958	NR_043536
27	Nereus interface	10%	*Halomonas meridiana*	99%	MG768959	AF212217
28	Erba sediment	18%	*Chromohalobacter israelensis*	98%	MG768971	NR_025431
29	Erba sediment	18%	*Salinivibrio siamensis*	99%	MG770368	NR_041552
30	Erba sediment	18%	*Idiomarina seosinensis*	99%	MG770369	NR_025826
31	Erba sediment	18%	*Pseudoalteromonas carrageenovora*	99%	MG770359	NR_026220
32	Erba sediment	18%	*Pseudoalteromonas ruthenica*	99%	MG770372	NR_025140
33	Erba sediment	18%	*Idiomarina baltica*	99%	MG770373	NR_027560
34	Erba interface	10%	*Pontibacillus halophilus*	99%	MG770364	NR_044532
35	Erba interface	10%	*Alteromonas macleodii*	99%	MG770376	Y18228
37	Nereus interface	10%	*Donghicolae burneus*	99%	MG770367	NR_043928
38	Nereus interface	10%	*Halomonas aquamarina*	99%	MG770380	NR_042063
39	Discovery interface	15%	*Idiomarina loihiensis*	100%	MG770382	NR_025119
40	Discovery interface	15%	*Idiomarina zobellii*	98%	MG770370	NR_024892
41	Nereus interface	10%	*Halomonas shengliensis*	99%	MG770371	NR_044099
42	Nereus interface	10%	*Vibrio communis*	99%	MG770378	GU078673
43	Nereus interface	10%	*Bacillus halodurans*	99%	MG770383	NR_025446
44	Nereus interface	10%	*Vibrio natriegens*	99%	MG770400	NR_026124
45	Nereus interface	10%	*Alteromonas macleodii*	98%	MG770410	AM885870
46	Nereus interface	10%	*Thalassospira tepidiphila*	100%	MG770411	NR_041492
47	Kebrit brine	26%	*Halostagnicola alkaliphila*	99%	MG775659	AB533256
53	Nereus interface	10%	*Salegentibacter mishustinae*	99%	MG770459	NR_025820
54	Nereus interface	10%	*Marinilactibacillus psychrotolerans*	99%	MG770461	NR_024794
55	Erba interface	10%	*Alcanivorax dieselolei*	99%	MG773786	NR_043106

### PCR amplification

DNA extraction, PCR amplification, and bioinformatics analyses of 16S rRNA genes from biomass of bacterial strains was performed according to Sagar et al. [7]. Sequences were deposited in Genbank, and accession numbers are listed in Table 1.

### Bacterial biomass

The inocula were streaked using three different media types as described by (Sagar et al. 2013 [6, 7]). These solidified media types were supplemented with either 10% or 15% or 20% or 26% NaCl (w/v) before autoclaving to mimic salt concentrations in their original habitat. All incubations were done in a Binder incubator (Type BD53, Binder, Tuttlingen, Germany). Strains reported here were isolated under oxic conditions (air, 21% O_2_) and atmospheric pressure (1 atm) described elsewhere [16, 17]. To collect biomass, all strains were grown for 2 to 3 weeks with constant agitation at 30 °C in 5.0 l of Marine Broth (Difco) supplemented with the respective concentration of NaCl. Bacterial biomass was harvested and ethyl acetate extracts were prepared according to Sagar et al. [6, 7].

### Cell culturing

BJ (Fibroblast), HCT (Colorectal adenocarcinoma), HT-1080 (Fibrosarcoma), MCF-7 (Breast Adenocarcinoma), IMR-32 (Neuroblastoma), BT20 (Breast Adenocarcinoma), and HeLa (Cervical carcinoma) were obtained from the American Type Cell Culture Collection (ATCC, Manassas, VA). The cells were cultured in DMEM (Dulbecco's Modified Eagle's Medium) containing 10% FCS (Foetal calf serum), and streptomycin (100 μg/mL) and penicillin (100 U/mL) in a 37 °C incubator supplying 5% CO_2_.

### MTT assay

2.5 × 10^3^ cells were seeded per well in 384-well culture plates and treated with 200 μg/mL marine bacterial extracts for 48 h. Growth inhibitory effects of extracts were estimated by an MTT (3-(4, 5-Dimethylthiazol-2-yl)-2, 5-diphenyltetrazolium bromide) assay as previously described [7]. A microtiter plate reader (BMG LabtechPHERAstar FS, Germany) was used to measure the OD (optical density) at 595 nm and the results were analyzed using Microsoft Office Excel©.

### APOPercentage assay

Cells were seeded in quadruplicates in 96 well plates at a density of 5 × 10^3^ cells per well in 90 μL of media. After 24 h, 200 μg/mL extracts were added to the cells for 48 h with, while 30 min treatment with 10 mM H_2_O_2_ was used as a positive control. The cells were lifted and stained with the APOPercentage dye (Biocolor, UK), and analysed as described previously [18].

### Caspase-3/7 activity assay

2.5 × 10^3^ cells were seeded in 20 μL of media in 384-well plates and allowed to settle overnight. Five microliters of extract (200 μg/mL) was added and further incubated for 48 h. Manufacturer’s instructions were followed to estimate Caspase-3/7 activity by using ApoTox-Glo kit (Promega) and the luminescence was measured using BMG LabtechPHERAstar FS (Germany). The results were normalized to cell viability (measured using MTT assay).

### Statistical analysis

The samples (untreated vs. treated) were compared by using Student’s *t*-test and statistical significance was noted at *p* < 0.05. A Z-score of ≥0.6 indicated robustness of assays [15].

## Results

### Taxonomic classification of microbes isolated from the Red Sea

Most bacteria isolated from the four brine pools Erba, Discovery, Kebrit, and Nereus were closely related to known and well described halophilic species within *Proteobacteria* and *Archaea* (Table 1).

### Anticancer activities of isolates in a panel of cell lines

A total of 60 extracts isolated from bacterial cultures from the four brine pools Erba, Discovery, Kebrait, and Nereus were screened for anticancer activity by determining cell growth inhibition through MTT assay (Table 2). Extracts induced varying levels of growth inhibition and were classified into five groups - empty circle (< 30%), quarter-moon (> 30 and < 40%), half-moon (> 40 and < 60%), three-quarter-moon (> 60 and < 80%) and full-moon (> 80%). The skin fibroblast cell line BJ was used as a normal cell line control for screening anticancer activity of the extracts. BJ cell growth was inhibited by approximately 50% of extracts while the growth of neuroblastoma cell line IMR-32 remained largely insensitive to the treatment. Interestingly, the growth of the triple negative breast cancer cell line BT20 was sensitive to the majority of the extracts.

**Table 2:**
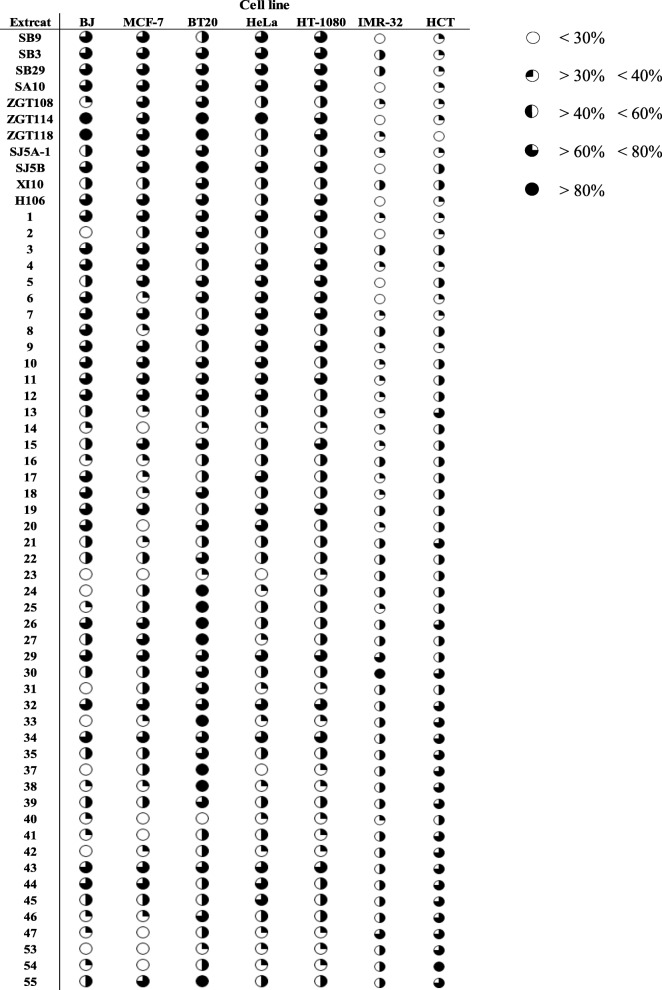
The percentage growth inhibition of various cell lines after treatment with extracts. Growth inhibition of one normal (BJ) and six cancer cell lines treated with 200 μg/ml microbial ethyl-acetate extract for 48 h

**Fig. 1:**
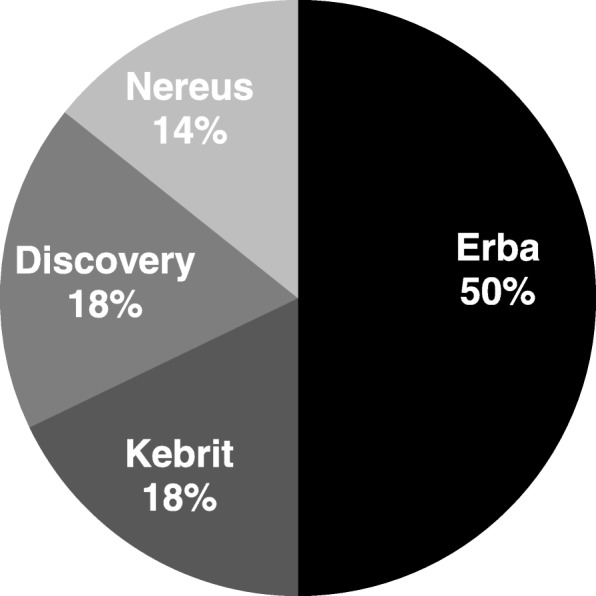
The percentage of active extracts from bacterial strains isolated from the respective brine-pools. Pie chart represents the distribution of extracts based on their source in the Red Sea that induced greater than 30% growth inhibition in various cancer cell lines at a concentration of 200 μg/ml after 24 h of treatment

Further analysis revealed that the majority of bacterial isolates inducing active growth inhibition (above 30%) in this study set were isolated from the Erba brine pool (Fig. 1). Figure 1 represents the distribution of active bacterial extracts inducing greater than 30% growth inhibition found across the brine-pools.

### Apoptosis as mode of anticancer activity

We assessed phosphatidylserine exposure in cancer cells treated with selected microbial extracts using APOPercentage assay and monitored change in caspase-3/7 activity to determine if extracts induced apoptosis. For this purpose, the extracts inducing > 30% growth inhibition were selected for apoptosis screening (Table 3). Due to the availability of limited amount of microbial extracts, we performed apoptosis and caspase-3/7 activity assays only on extracts active in breast cancer, cervical cancer, and fibrosarcoma cell lines. Again, BJ cells were used as a control to identify extracts with selective anticancer activity. Extracts induced apoptosis in all cell lines. However four (ZGT118, XI10, 7 and 13), six (ZGT118, XI10, 13, 16, 30 and 55), one (9), and three (10, 15 and 22) extracts selectively induced apoptosis in MCF-7, BT20, HeLa and H T-1080 cells compared to BJ cells, respectively. The extract number 7 was only active against MCF-7 cells, whereas extracts 16 and 55 were selectively active against BT20 cells when the apoptosis-inducing potential of these extracts was compared among all five cell lines (Table 3).

**Table 3:**
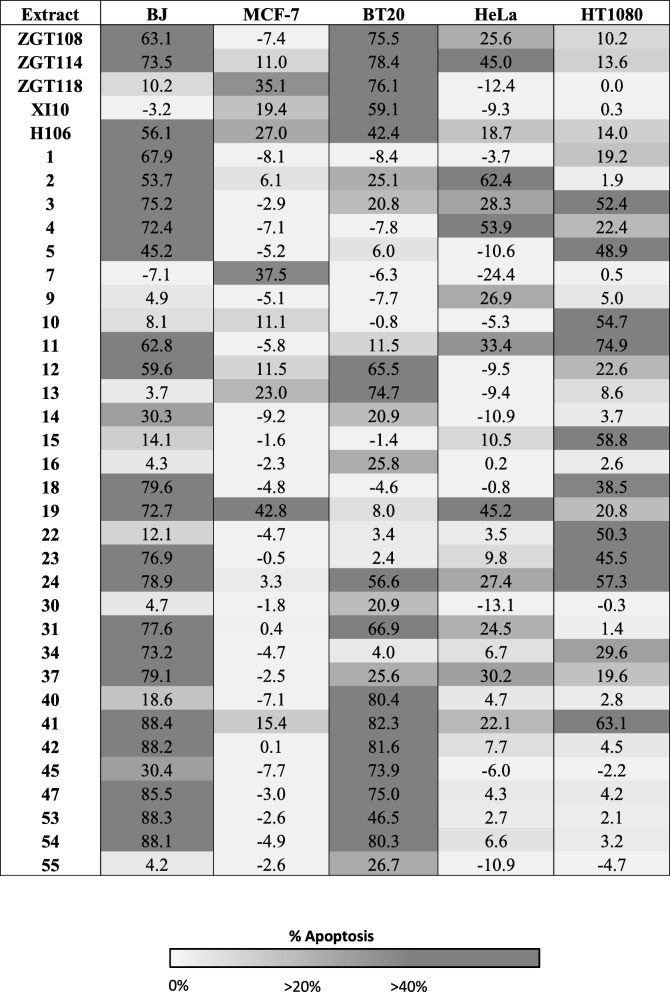
The percentage apoptotic cell death induced by selected extracts in various cell lines. Heat map of extracts (200 μg/ml) inducing apoptosis in one normal (BJ) and selected cancer cell lines after 48 h treatment

**Fig. 2:**
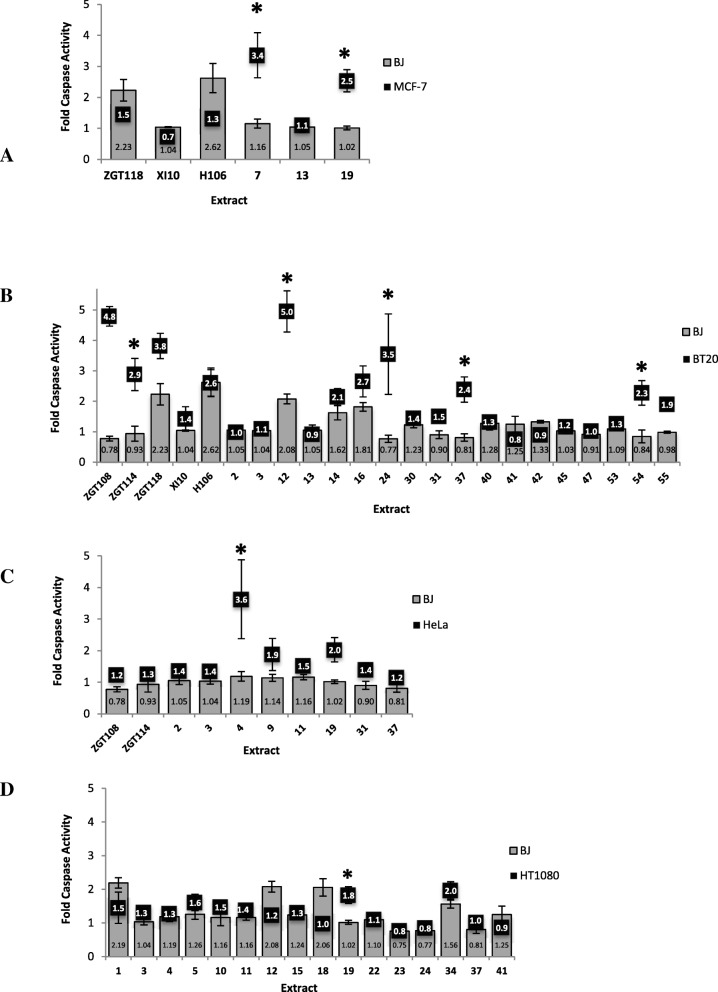
Caspase 3/7 Activity in cells treated with bacterial extract. Normal skin fibroblast cell line BJ and four cancer cell lines MCF-7, BT20, HeLa and HT1080 were treated with 200 μg/ml bacterial extract for 48 h (**a**-**d**). Fold change in Caspase-3/7 activity relative to untreated was determined as per manufacturer’s instructions and students t-test was used to determine significance where * indicates *p* < 0.05

A Caspase-3/7 activity assay was employed to gain insights into the type of apoptosis occurring, namely caspase-dependent or caspase-independent (Fig. 2a-d). The caspase-3/7 results showed a clear trend that a caspase-independent mechanism was mostly responsible for BJ cell death. Extract number 7 induced caspase-dependent apoptosis in MCF-7 cells but had no adverse effect on BJ cell death despite it inducing slight growth inhibition in BJ cells making it a promising drug candidate for future work. Extract number 19 induced caspase-dependent apoptosis in MCF-7, HeLa, and HT1080 cells but not in BJ cells, even though BJ cells stained positive for phosphatidylserine exposure by APOPercentage assay (Table 3). Caspase-3/7 activity increased in BT20 cells compared to BJ cells in response to extracts ZGT108, ZGT114, 12, 24, 37, 54 and 55. HeLa cells and HT1080 cells displayed significantly increased caspase-3/7 activation in response to extracts 4 and 19, respectively (Fig. 2). Interestingly, extract number 55 showed selectivity towards BT20 cells by inhibiting its growth and inducing apoptosis via caspase-3/7, neither of which was observed for BJ cells.

## Discussion

As a follow-up from our previous studies [6, 7], we screened extracts from 60 marine bacteria isolated from brine pools of the Red Sea. We would like to emphasize that in our previous two investigations, we could only find extracts active against MCF-7 (ER+) cells, but the current study reports anticancer activities of the extracts isolated from the Red Sea brine-pool harboring bacteria against fibrosarcoma, cervical cancer and particularly BT20 (triple negative) cancer cells. Primary cytotoxicity screening of all extracts against seven cell lines representing five different cancers enabled us to broadly identify potential anticancer extracts from deep-sea microbes. All extracts were also screened against one normal fibroblast cell line (BJ) to further identify those who exhibited selectivity for cancer, but not normal cells.

We investigated whether the brine-pool location had any correlation with anticancer activity induced by the bacterial isolates. Of all isolates from the respective brine-pools, we found that about 50% of all isolates collected from Erba Deep (located at a depth of 2395 m), showed greater than 30% growth inhibition (Fig. 1). The most closely related validly described species to these strains are (Table 1): *Chromohalobacter israelensis, Salinivibrio siamensis, Idiomarina seosinensis, Pseudoalteromona scarrageenovora, Pontibacillus halophilus, and Alteromonas macleodii.* Our previous work [7] has reported anticancer activity of *Chromohalobacter israelensis* in HeLa cells*.* A PubMed search did not reveal even a single article that describes the anticancer activity of any of the other five bacterial species. This highlights the fact that the microbes found in the deep-sea brine pools of the Red Sea (especially Erba Deep) may have unique anticancer compounds that can be explored in the future to develop new drugs.

Apoptosis assays (APOPercentage and caspase-3/7 activity) confirmed that several of these selected (showing > 30% cell growth inhibition) extracts induced apoptotic cell death in cancer cell lines. This secondary screening process identified the extracts that selectively targeted a specific type of cancer via apoptotic cell death. We further investigated if extracts from a particular bacterial species have anticancer activity towards a specific cell line. Intriguingly, all three extracts (10, 15 and 22) that specifically inhibited the growth of fibrosarcoma cells (HT-1080) belonged to strains that were closely related to *Salinivibrio costicola, Salinivibrio sharmensis,* and *Salinicola salaries,* respectively, showing an enrichment of genus *Salinivibrio.* It would be interesting in future to investigate *Salinivibrio* extracts against other sarcomas as well. In MCF-7 cells, two out of four most active extracts (XI10 and 13) belonged to *Pseudoalteromonas mariniglutinosa.* This bacterial species had not been tested so far for anticancer activities (PubMed search). Secondary metabolites isolated from *Psuedoalteromonas* sp. off the coast of Brazil were reported to have potent anticancer activity against a leukemic and melanoma cell line, and the active compound, prodigiosin, was shown to be selective for cancers overexpressing ErbB-2 [19]. In BT20 cells, extracts (16 and 55) showed selective cell death, and these extracts belong to species *Halomonas hamiltonii* and *Alcanivorax dieselolei*, respectively, none of which have ever been shown to have anticancer activity before. Our observation that *Halomonas* sp. and *Alteromonas* sp. contributed to the majority of growth inhibition may be attributed to their ability to produce molecules including exopolysaccharides (EPSs) and Dithiolopyrrolone (DTP), respectively. Exopolysaccharides (heterogeneous polymers) isolated from *Halomonas stenophila* and *Halomonas smyrnensis* induced pro-apoptotic effects against human T-leukemia cells [20] and breast cancer MCF-7 cells [21]. *Alteromonas* sp. are also well known for producing dithiolopyrrolone (DTP) molecules, which are known potent natural antibiotics; DTP obtained FDA approval as topical antibiotic Bactroban® (GlaxoSmithKline) [22]. Not surprising, DTP also exhibits potent anticancer activity. These classes of antibiotic or polysaccharide type molecules most probably explain the anticancer activity we observed in this study.

In conclusion, our study has identified several microbial species that have the potential to kill selectively cancer cells, and interestingly many of these species have never been previously tested for their anticancer activities. Here, we provide seminal baseline data pinpointing which bacterial species and brine-pools should be targeted for future investigations to isolate anticancer compounds. This work is of particular importance for triple negative breast cancer therapeutic development as no drug exists till date that can effectively cure this aggressive form of breast cancer. Testing of these marine extracts against penta-negative breast cancer cells should be of great interest to future studies.

## Data Availability

All 16S rRNA gene sequences of newly isolated strains from this study have been deposited in Genbank. Please refer to Table 1 for accession numbers.

## Abbreviations

DMEM: Dulbecco’s Modified Eagle’s Medium
DTP: Dithiolopyrrolone
ER: Estrogen receptor
FCS: Foetal calf serum
MTT: 3-(4, 5-Dimethylthiazol-2-yl)-2, 5-diphenyltetrazolium bromide
OD: Optical density
PS: Phosphatidylserine
